# Influence of population mobility on the novel coronavirus disease (COVID-19) epidemic: based on panel data from Hubei, China

**DOI:** 10.1186/s41256-020-00151-6

**Published:** 2020-06-08

**Authors:** Junfeng Jiang, Lisha Luo

**Affiliations:** 1 School of Health Sciences, Wuhan University, No.115 Donghu Road, Wuhan, 430071 China; 2 Center for Evidence-based and Translational Medicine, Zhongnan Hospital of Wuhan University, Wuhan, China; 3 Department of Evidence-Based Medicine and Clinical Epidemiology, the Second Clinical College of Wuhan University, Wuhan, China

**Keywords:** COVID-19, Population mobility, Infectious disease epidemic, Lockdown intervention

## Abstract

**Background:**

The novel coronavirus disease (COVID-19) was first reported in Wuhan, China. The mass population mobility in China during the Spring Festival has been considered a driver to the transmission of COVID-19, but it still needs more empirical discussion.

**Methods:**

Based on the panel data from Hubei, China between January 6th and February 6th, 2020, a random effects model was used to estimate the impact of population mobility on the transmission of COVID-19. Stata version 12.0 was used, and *p* < 0.05 was considered statistically significant.

**Results:**

The COVID-19 was more likely to be confirmed within 11–12 days after people moved from Wuhan to 16 other prefecture-level cities in Hubei Province, which suggests a period of 11–12 days from contact to being confirmed. The daily confirmed cases and daily increment in incidence in 16 prefecture-level cities show obvious declines 9–12 days post adaptation of city lockdown at the local level.

**Conclusion:**

Population mobility is found to be a driver to the rapid transmission of COVID-19, and the lockdown intervention in local prefecture-level cities of Hubei Province has been an effective strategy to block the COVID-19 epidemic.

The novel coronavirus disease (COVID-19) was first reported in December, 2019 in Wuhan and has been rapidly spreading to other areas in China since then [1, 2]. On January 20th, 2020, the first confirmed case in Huanggang, Hubei was reported; on January 22nd, some confirmed cases were also reported in Jingzhou and Jingmen in Hubei; on January 27th, all prefecture-level cities in Hubei reported confirmed cases of COVID-19. By the end of February 29th, a total of 66,907 confirmed cases of COVID-19 and 2761 deaths directly caused by the COVID-19 had been reported in Hubei Province [3]. Given the high infectivity of COVID-19, the Chinese central government has attached great importance to it. Wuhan closed all its routes leaving Wuhan and suspended its public transportation at 10 a.m. on January 23rd, 2020. Within 2 days after that, 14 other prefecture-level cities in Hubei Province also successively announced their lockdown policies to limit population mobility. By January 25th, there had been 26 provinces that had launched the first-level response to major public health emergencies, covering a total population of more than 1.2 billion in China.

The COVID-19 is reported to spread mainly through respiratory droplets, direct contact, aerosol diffusion and so forth [4–6]. The transmission of such airborne diseases is closely related to population mobility, because pathogens in the air may spread along the path of population mobility during the transmission of such viruses [7]. For example, some epidemiological studies on the severe acute respiratory syndrome (SARS) report that population mobility can spread the SARS epidemic to other areas [8], and the area along expressways or near interprovincial expressways has the highest risk of SARS infection [9]. Studies on the transmission mechanism of tuberculosis show that Chinese internal migrants can bring tuberculosis virus back to their hometown and cause a rapid transmission of tuberculosis [10]. Evidence from influenza also suggests that a close proximity to other people during population mobility (e.g., travelling on the same train for a long time with people with influenza) greatly increases the chance of influenza infection [11]. Also, because the transmission ways or mechanisms between the COVID-19 and the airborne diseases above are similar, the mass population mobility during the Spring Festival in China (also called *Chunyun*) may be a driver to the spread of COVID-19. The rush of people brought by *Chunyun* has greatly increased the population density in railway and passenger stations, which has also increased the difficulty for governments and health institutions in controlling the transmission of COVID-19 [6].

Some recently published studies have also discussed the transmission of COVID-19 and whether it related to population mobility. For example, based on the real-time mobility data from Wuhan, Kraemer et al. found that the drastic control measures implemented in China, including travel restrictions and social distancing, substantially mitigated the spread of COVID-19, because less confirmed cases were reported to have experiences of traveling to Wuhan 1 week after its lockdown intervention [12]. Pan et al. predicted that the lockdown intervention in China reduced the number of confirmed cases by 96%, and the lockdown of Wuhan delayed the spread of the COVID-19 epidemic to other cities by at least 2.91 days [13]. Using a second derivative model, Chen and Yu found that although the COVID-19 epidemic displayed a nonlinear and chaotic feature, it showed a decline within 14 days after massive interventions had been conducted, which indicated an incubation period of 14 days for COVID-19 [14]. Yang et al. used a modified susceptible-exposed-infectious-removed (SEIR) model to examine the effect of lockdown conducted in January on the size of the COVID-19 epidemic; they found the lockdown of Wuhan significantly reduced the number of confirmed cases, and it might be three times larger if Wuhan’s lockdown intervention was carried out 5 days later [15]. Similarly, based on a time-delay dynamic system model, Yan et al. thought that if the mass population mobility emerged again in February, the COVID-19 epidemic would be more severe and was harder to control [16]. In addition to China, similar evidence that travel restrictions and social distancing caused by the lockdown intervention mitigate the spread of COVID-19 is also found in other countries [17–20], indicating a positive effect of population mobility on the transmission of COVID-19 more or less.

The World Health Organization (WHO) reported that China’s intensive public health interventions, including lockdown and social distancing, have significantly contributed to the containment of COVID-19 [21]. Accordingly, the widespread transmission of COVID-19 in China may be highly related to the mass population mobility attributable to *Chunyun* in 2020. However, this issue needs to be further discussed, as it is still unclear to what extent the mass population mobility during *Chunyun* has influenced the COVID-19 epidemic, and the exact association between population mobility and the COVID-19 epidemic is also unclear. There is no doubt that examining the impact of population mobility on the COVID-19 epidemic will help us understand how the lockdown intervention that aims to reduce population mobility can help us fight the epidemic. Based on the data from Hubei Province, the present study aimed to estimate the influence of population mobility on the transmission of COVID-19.

## Data and methods

### Data sources

The data used in this study were collected from multiple sources that are open-access. First, data on confirmed COVID-19 cases in 16 prefecture-level cities of Hubei Province (except for Wuhan) from January 22nd to February 6th, 2020 were obtained from the official website of the Health Commission of Hubei Province.^1^ Second, the proportion of Wuhan’s outflow population that flowed into 16 other prefecture-level cities in Hubei Province was collected from the Baidu Migration Big Data Website. This aggregated mobility data can help us understand population mobility status during this era [22] and supports this study. It is reported that the incubation period of COVID-19 is 3–7 days, with a maximum of 14 days [23]. Accordingly, we collected data on population mobility from January 6th, 2020 (16 days ahead of January 22nd, 2020).^2^ Although Wuhan was locked down on January 23rd, there were still a large number of people leaving Wuhan on this day and the following 2 days due to the Spring Festival and many other reasons. Therefore, the deadline for the data collection of population mobility was set as January 25th, 2020 in this study. Third, the spatial distances between Wuhan and 16 other prefecture-level cities in Hubei Province were measured using the Baidu Map. Fourth, the time of city lockdown in 16 prefecture-level cities of Hubei Province was obtained from the official website of local governments. Finally, the socio-economic development variables used in this study were obtained from the *Statistical yearbooks of Hubei Province* or 16 prefecture-level cities in 2018 or 2019.

There were two reasons for choosing 16 prefecture-level cities in Hubei Province as our research objects. First, the COVID-19 was first reported in Wuhan, and Wuhan has close economic ties with other prefecture-level cities in Hubei Province. During the Spring Festival in 2020, about 70.0% of the outflow population of Wuhan directly flows into 16 other prefecture-level cities in Hubei Province (see Appendix 1). Therefore, data on confirmed COVID-19 cases in 16 prefecture-level cities in Hubei Province were good materials for investigating the primary infection (spread from Wuhan to other areas) of COVID-19. Second, compared with the large gap in natural conditions and social-economic development among provinces in China, there was less heterogeneity among 16 prefecture-level cities in Hubei Province in terms of the natural environment and social-economic development, so they were more suitable for the statistical analysis in this study.

### Variables

#### Outcomes

**Fig. 1:**
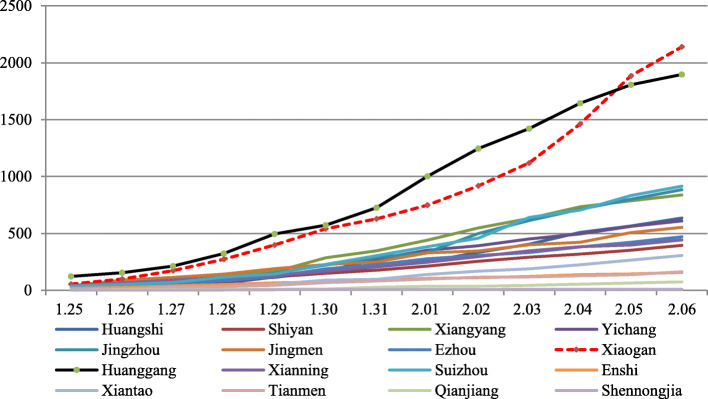
Cumulative number of confirmed COVID-19 cases in 16 prefecture-level cities in Hubei Province from January 25th to February 6th, 2020

**Fig. 2:**
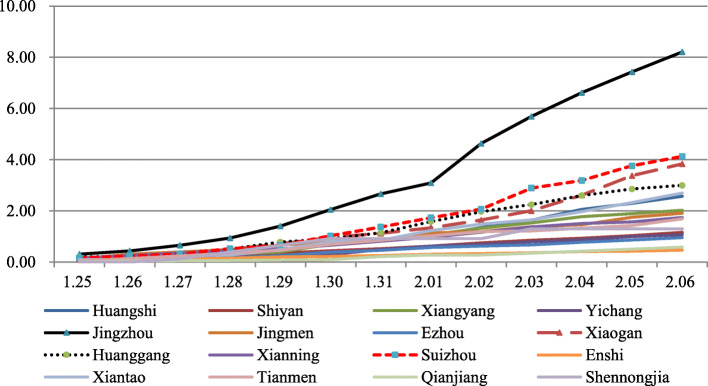
Cumulative diagnosis rate per 10,000 persons of COVID-19 in 16 prefecture-level cities in Hubei Province from January 25th to February 6th, 2020

The number of daily confirmed COVID-19 cases in 16 prefecture-level cities in Hubei Province (except for Wuhan), the proportion of daily confirmed COVID-19 cases in 16 prefecture-level cities in the total daily cases in Hubei Province, and the diagnosis rate of COVID-19 per 10,000 persons in 16 prefecture-level cities in Hubei Province were used as our outcomes. More details can be seen in Figs. 1 and 2.

#### Explanatory variables

To reflect the population mobility from Wuhan to 16 other prefecture-level cities in Hubei Province, a specific lag of proportion of the population moving from Wuhan to each prefecture-level city in Hubei Province was used as the first explanatory variable. We should consider the period from contact to being confirmed (contact-infection-latency-onset-diagnosis), and this period will be longer than the incubation period (3–7 days in most cases).

The city lockdown was an important intervention or policy to restrict population mobility after the outbreak of the COVID-19 epidemic. In this study, the date of lockdown in 16 prefecture-level cities (see Appendix 2) was used to estimate the effect of lockdown on the transmission of COVID-19. The differences in lockdown policies among different cities were difficult to quantify, so its potential impacts were not considered in this study.

#### Control variables

This study focused on the COVID-19 epidemic related to population mobility from Wuhan to other prefecture-level cities in Hubei Province (primary infection). Therefore, some factors associated with secondary infection (e.g., community infection) need to be controlled for. Accordingly, economic development, richness of health resources, population density and distance to Wuhan were used as our control variables. Per capita gross domestic product (GDP) in 2018 was used to measure economic development; the number of medical and health institutions’ beds and healthcare workers per thousand persons in 2017 were used to measure health resources richness; the population density of each prefecture-level city in 2018 was used; distance to Wuhan was measured using the ranging tool in the Baidu Map, and an average value of three measurements was used to reduce potential measurement errors. Details can be seen in Appendix 2.

## Methods

In this study, descriptive analysis was used to display the time series of confirmed COVID-19 cases from January 25th to February 6th in Hubei Province. The time series line chart was also used to present daily confirmed cases and analyze the influence of city lockdown on the transmission of COVID-19; this strategy can also be called a first derivative model, which is similar to the strategy used in Chen and Yu’s study [14].

The random effects (RE) model was used to estimate the impact of population mobility on the transmission of COVID-19.^3^ Because the infection, incubation and diagnosis of COVID-19 will cost some time, the lag term of population mobility was set as our explanatory variable. It is reported that the incubation period of COVID-19 is 3–7 days, with a maximum of 14 days [23]. Considering the duration from contact to being confirmed, a lag period of 5–16 days was used. The estimated coefficient and R-square value were reported to help determine the best fitting model.

In this study, Microsoft Excel 2016 was used to draw figures, the RE model was performed using Stata version 12.0 [24]. *p* < 0.05 was considered statistically significant.

## Results

### Descriptive analysis

Figure 1 shows that there had been 2141 confirmed cases in Xiaogan and 1897 confirmed cases in Huanggang as of February 6th, 2020, these two cities had the largest number of confirmed COVID-19 cases in Hubei Province (except for Wuhan). Although the cumulative confirmed cases in Huanggang ranked first before February 4th, the growth rate of new daily confirmed cases decreased significantly after February 2nd, so it retired to the second place after February 5th. The total number of confirmed cases in Xiangyang, Suizhou and Jingzhou was almost the same, which had reached 800–900 as of February 6th. Qianjiang and Shennongjia had the fewest cumulative confirmed cases (less than 100 as of February 6th). Appendix 1 shows that the proportion of the inflow population in 16 prefecture-level cities from Wuhan was highly correlated with the number of confirmed cases, reflecting a close association between mass population mobility during *Chunyun* and the transmission of COVID-19.

Figure 2 shows that Jingzhou had the highest cumulative diagnosis rate of COVID-19 on February 6th (8.21‱), followed by Suizhou (4.13‱), Xiaogan (3.83‱), Huanggang (3.00‱) and Xiantao (2.69‱) in order; Enshi (0.46‱), Qianjiang (0.58‱) and Ezhou (0.96‱) had low cumulative diagnosis rates of COVID-19. It can be seen that the cumulative diagnosis rate of COVID-19 in all 16 prefecture-level cities rose continuously from January 25th to February 6th.

### Population mobility and transmission of COVID-19

Table 1 shows that, without other covariates controlled for, the R-square was the largest (0.332 in Panel A) when the lag period was 12 days; with other covariates controlled for, the R-square was still the largest (0.338 in Panel B) when the lag period was 12 days. That is to say, the COVID-19 was more likely to be confirmed within 12 days after people (had contact with COVID-19 cases) left Wuhan for other prefecture-level cities in Hubei Province. Thus, the duration from contact to being confirmed was mostly concentrated in 12 days, and every 1% increase in the proportion of population mobility averagely increased the proportion of confirmed COVID-19 cases by 1.395% (*p* < 0.001).

**Table 1:** Population mobility and proportion of confirmed COVID-19 cases: based on RE model

	Panel A			Panel B			N
	Coefficient	95%CI	R-square	Coefficient	95%CI	R-square	
Model 1: Lag 5 days	1.145***	1.003–1.288	0.198	1.049***	0.592–1.506	0.204	144
Model 2: Lag 6 days	1.175***	1.021–1.329	0.221	1.206***	0.788–1.624	0.227	160
Model 3: Lag 7 days	1.199***	0.984–1.414	0.241	1.226***	0.715–1.736	0.248	176
Model 4: Lag 8 days	1.220***	1.021–1.419	0.257	1.210***	0.813–1.606	0.264	192
Model 5: Lag 9 days	1.230***	1.041–1.418	0.267	1.176***	0.776–1.576	0.275	208
Model 6: Lag 10 days	1.283***	1.123–1.444	0.294	1.263***	0.888–1.638	0.301	224
Model 7: Lag 11 days	1.353***	1.253–1.454	0.327	1.444***	1.158–1.730	0.335	240
Model 8: Lag 12 days	1.354***	1.200–1.508	0.332	1.395***	0.989–1.801	0.338	256
Model 9: Lag 13 days	1.379***	1.265–1.493	0.326	1.384***	1.095–1.672	0.332	256
Model 10: Lag 14 days	1.430***	1.291–1.568	0.330	1.478***	1.144–1.812	0.336	256
Model 11: Lag 15 days	1.455***	1.311–1.600	0.325	1.486***	1.145–1.826	0.331	256
Model 12: Lag 16 days	1.468***	1.304–1.633	0.321	1.478***	1.089–1.867	0.327	256

Note: *p < 0.05, **p < 0.01, ***p < 0.001. CI: confidence interval. Robust standard error was used in the above models. No other covariate was controlled for in all models in Panel A; spatial distance to Wuhan, per capita GDP, the number of medical and health institutions’ beds and healthcare workers per thousand persons, and population density were controlled for in all models in Panel B

In Table 2, the outcome variable was changed from the proportion of confirmed cases to the number of confirmed cases to perform a robustness analysis. Without other covariates controlled for, the R-square was the largest (0.463 in Panel A) when the lag period was 12 days; by contrast, with other covariates controlled for, the R-square was the largest (0.487 in Panel B) when the lag period was 11 days. Every 1% increase in the proportion of population mobility averagely increased the number of confirmed cases by 15.184 (*p* < 0.001). This also suggests that the COVID-19 was more likely to be confirmed within 11–12 days after people left Wuhan for other prefecture-level cities in Hubei Province, which was almost in line with the results in Table 1.

**Table 2:** Population mobility and number of confirmed COVID-19 cases: based on RE model

	Panel A			Panel B			N
	Coefficient	95%CI	R-square	Coefficient	95%CI	R-square	
Model 1: Lag 5 days	4.192***	3.871–4.512	0.329	4.418***	2.676–6.160	0.351	144
Model 2: Lag 6 days	4.664***	4.080–5.248	0.347	5.219***	3.747–6.691	0.380	160
Model 3: Lag 7 days	5.758***	4.277–7.239	0.356	6.274***	4.252–8.296	0.397	176
Model 4: Lag 8 days	6.608***	4.964–8.252	0.368	7.074***	5.214–8.933	0.398	192
Model 5: Lag 9 days	7.510***	6.281–8.739	0.385	8.609***	6.416–10.802	0.412	208
Model 6: Lag 10 days	9.136***	8.383–9.888	0.433	12.166***	8.291–16.040	0.455	224
Model 7: Lag 11 days	10.600***	9.592–11.608	0.451	15.184***	9.591–20.777	0.487	240
Model 8: Lag 12 days	10.812***	8.932–12.693	0.463	14.996***	9.293–20.698	0.482	256
Model 9: Lag 13 days	11.005***	9.367–12.643	0.460	15.113***	10.035–20.190	0.480	256
Model 10: Lag 14 days	11.410***	10.028–12.792	0.458	16.171***	10.538–21.805	0.480	256
Model 11: Lag 15 days	11.584***	10.005–13.164	0.449	16.626***	9.724–23.527	0.472	256
Model 12: Lag 16 days	11.751***	9.861–13.641	0.444	17.086***	9.118–25.054	0.466	256

Note: *p < 0.05, **p < 0.01, ***p < 0.001. CI: confidence interval. Robust standard error was used in the above models. No other covariate was controlled for in all models in Panel A; spatial distance to Wuhan, per capita GDP, the number of medical and health institutions’ beds and healthcare workers per thousand persons, and population density were controlled for in all models in Panel B

### Influence of lockdown on the transmission of COVID-19

**Fig. 3:**
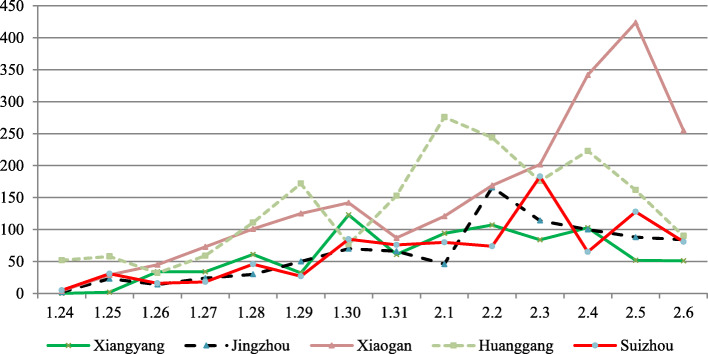
Daily confirmed cases of COVID-19 in 5 prefecture-level cities in Hubei Province from January 24th to February 6th, 2020

Also, because Xiaogan, Huanggang, Suizhou, Jingzhou and Xiangyang had the most cumulative confirmed cases on February 6th, 2020, we used Fig. 3 to display the daily confirmed COVID-19 cases per 10,000 persons in these five prefecture-level cities from January 24th to February 6th, 2020. It can be seen that the number of daily confirmed cases in Xiaogan sharply dropped for the first time on February 6th in 2020, corresponding to the 12th day of its lockdown (conducted at 0:00 on January 25th). The number of daily confirmed cases in Huanggang decreased significantly after February 2nd, corresponding to the 9th day of its lockdown (conducted at 0:00 on January 24th). The number of daily confirmed cases in Suizhou significantly decreased on February 4th, corresponding to the 10th day of its lockdown (conducted at 0:00 on January 25th). The number of daily confirmed cases in Jingzhou significantly decreased on February 3rd, corresponding to the 10th day of its lockdown (conducted at 0:00 on January 24th). The number of daily confirmed cases in Xiangyang significantly decreased on February 5th, corresponding to the 9th day of its lockdown (conducted at 0:00 on January 27th). These results were generally consistent with an 11–12 days lag period between population mobility and the diagnosis of COVID-19 estimated in Tables 1 and 2.

**Fig. 4:**
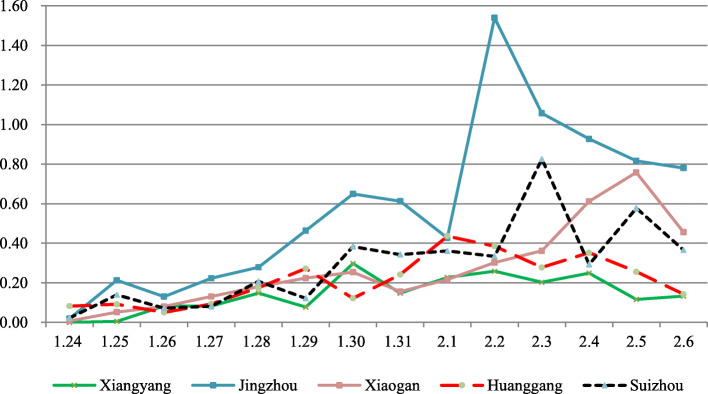
Daily diagnosis rate of COVID-19 per 10,000 persons in 5 prefecture-level cities in Hubei Province from January 24th to February 6th, 2020

Figure 4 shows that the daily increase of diagnosis rate per 10,000 persons in Jingzhou had continuously declined since February 3rd, corresponding to the 10th day of its lockdown. The daily increase of diagnosis rate in Suizhou had steadily declined since February 4th, corresponding to the 10th day of its lockdown. The daily increase of diagnosis rate in Xiaogan had significantly declined since February 6th, corresponding to the 12th day of its lockdown. The daily increase of diagnosis rate in Huanggang had begun to slowly decline since February 2nd, corresponding to the 9th day of its lockdown. The daily increase of diagnosis rate in Huanggang had begun to slowly decline since February 5th, corresponding to the 9th day of its lockdown. These results were also generally consistent with an 11–12 days lag period between population mobility and the diagnosis of COVID-19 estimated in Tables 1 and 2.

## Discussion

Based on the data from Hubei Province, this empirical study comprehensively estimated the relationship between population mobility and the transmission of COVID-19. We found that there was a period of 11–12 days from contact to being confirmed for most infected COVID-19 cases in Hubei Province (except for Wuhan). This was highly in line with the situation that the daily increase in confirmed cases and diagnosis rate began to show significant declines within 9–12 days after the lockdown of 16 prefecture-level cities. The mass population migration during the Spring Festival in 2020 has motivated the transmission of COVID-19, and the lockdown of Wuhan and other cities in Hubei Province has effectively mitigated the COVID-19 epidemic.

The duration from contact to being confirmed for most COVID-19 cases in Hubei Province was reported to be 11–12 days in this study, which far exceeds the reported 3–7 days incubation period. This is highly in line with the results from Chen and Yu’s as well as Kraemer et al.’s studies (about 14 days) [12, 14]. It does not mean that population mobility only has an exact lagging effect (11–12 days) on COVID-19 transmission; by contrast, it indicates the closest relationship between them, and other time lags (e.g., lag-13 days) also matter but are not the most important. Around 9–12 days after the lockdown of 16 prefecture-level cities in Hubei Province (except for Wuhan), the daily increase of confirmed COVID-19 cases showed a significant decline. Some prior studies indicate that the lockdown of Wuhan and other areas was conductive to the control of the COVID-19 epidemic [12, 15, 16]. Specific lockdown interventions, such as travel restrictions and social distancing, are mainly used to reduce population mobility and can help mitigate the COVID-19 epidemic [25], so our results support the opinion that population mobility is closely related to the transmission of COVID-19 [12, 13, 26, 27].

It should be noted that this 11–12 days period should not be simply considered as the incubation period, but the duration from contact/infection to being confirmed. Most COVID-19 cases in Hubei Province spend 11–12 days from contact/infection to being confirmed, and it particularly reflects a long time from onset to being confirmed and low diagnostic efficiency. On the one hand, Hubei Province faced a shortage of medical and health resources when facing this major public health emergency in the first 3 months of 2020. The number of confirmed COVID-19 cases in Hubei Province had exceeded 60,000 as at February 29th, which accounted for more than 70% of the total confirmed cases in China. This emergency is a challenge to the health service capacity of Hubei Province. The Chinese central government required other neighboring provinces to support Hubei to control the COVID-19 epidemic as soon as possible [28], and this intervention has achieved a good result. On the other hand, in the first 2 months of 2020, the production and supply of nucleic acid detection reagents were severely inadequate in Hubei Province, which may have delayed the diagnosis of some COVID-19 cases.

Nevertheless, this study also found some positive information. Most prefecture-level cities in Hubei Province began to lock down on January 23rd, 24th or 25th, and some cities with severe epidemics in other provinces (e.g., cities in Zhejiang, Henan, Guangdong, and Anhui) also began to implement similar policies in later January or early February to restrict population mobility. According to the roughly estimated turning point of the COVID-19 epidemic in Hubei Province (9–12 days), a nationwide restriction on population mobility can help block the transmission of COVID-19. The fact is that, the turning point of the COVID-19 epidemic in other provinces appeared on around February 14th, which is 10–15 days after their lockdown interventions. Because the lockdown intervention is mainly used to reduce population mobility, the fact that the turning point in most provinces and cities emerged within 10–15 days after their lockdown supports our results more or less. This result is also in line with the opinion that lockdown interventions are most useful in the early and late stages of COVID-19 outbreaks [12]. Furthermore, our findings also support or justify the national policy of requiring a 14-day quarantine before work can resume.

There are also some limitations to this study. First, we have no access to the data on the number of persons that traveled from Wuhan to other prefecture-level cities in Hubei Province, so it is hard for us to exactly predict the size of the COVID-19 epidemic, and we only yielded the best fitting lag length of 11–12 days and concluded that the city lockdown in Hubei could help control the epidemic. Second, the daily confirmed COVID-19 cases reflect the cases detected rather than the cases infected, so there may be measuring errors because not every COVID-19 case can be immediately confirmed. In this case, the influence of population mobility on the COVID-19 epidemic may be underestimated. Finally, many other factors were not considered in this study when analyzing the impact of city lockdown. For example, the COVID-19’s prevention and control measures in many regions have been gradually upgraded, which also greatly promotes the arrival of the turning point of the epidemic. However, the sample size of this study was relatively small, the statistical power was moderate, and some confounding factors were hard to control. Therefore, although the results from the RE model (11–12 days) and the first derivative model (9–12 days) are similar, the judgment of the turning point of the epidemic only has moderate reference value and should be treated with caution.

## Conclusions

This study investigated the influence of population mobility on the transmission of COVID-19. We found that the COVID-19 was more likely to be confirmed within 11–12 days after people moved from Wuhan to other cities in Hubei Province, which suggests a period of 11–12 days from contact to being confirmed. Also, the daily confirmed cases and daily increment in incidence in 16 prefecture-level cities show obvious declines 9–12 days post adaptation of city lockdown. Population mobility is found to be a driver to the transmission of COVID-19, and the city lockdown started from January in Hubei Province has effectively mitigated the COVID-19 epidemic. Thus, strategies such as social distancing and travel restrictions, as well as quarantine no less than 12 days, should be encouraged to fight the COVID-19 pandemic.

## Data Availability

No additional data are available.

## Appendix 1

**Table 3:** Proportion of population moving from Wuhan to 16 prefecture-level cities in Hubei Province (%)

Cities	Date									
	1.6	1.7	1.8	1.9	1.10	1.11	1.12	1.13	1.14	1.15
Huangshi	3.71	3.70	3.57	3.43	3.42	3.81	3.74	3.70	3.69	3.68
Shiyan	1.98	2.04	1.87	1.89	2.02	1.85	1.88	1.76	1.65	1.60
Xiangyang	4.04	4.28	4.32	4.15	4.12	3.92	3.66	3.72	3.68	3.44
Yichang	2.75	2.89	2.85	2.77	3.08	3.24	2.76	2.76	2.43	2.35
Jingzhou	5.52	5.83	5.95	5.81	5.74	5.91	5.74	5.80	5.84	6.03
Jingmen	2.63	2.74	2.76	2.70	2.85	2.95	2.72	2.76	2.73	2.82
Ezhou	5.23	4.60	4.06	3.93	4.12	4.53	4.83	4.77	4.36	4.10
Xiaogan	11.04	10.68	10.41	10.99	10.94	13.00	13.47	12.04	12.61	13.14
Huanggang	10.92	10.81	10.77	11.02	10.52	11.75	11.19	11.39	12.55	13.30
Xianning	5.06	4.74	4.95	4.66	5.22	5.95	5.32	4.94	4.97	5.10
Suizhou	2.55	2.55	2.59	2.65	2.52	2.71	2.65	2.67	2.68	2.82
Enshi	1.94	2.03	2.11	2.29	2.12	1.92	2.11	1.83	1.89	1.79
Xiantao	2.30	2.31	2.29	2.33	2.38	2.81	2.80	2.66	2.59	2.88
Tianmen	1.43	1.59	1.60	1.54	1.47	1.76	2.01	1.77	1.95	1.97
Qianjiang	1.07	1.16	1.08	1.18	1.10	1.14	1.28	1.18	1.04	1.01
Shengnongjia	0.00	0.00	0.00	0.00	0.00	0.11	0.12	0.00	0.00	0.00
Total	62.33	62.13	61.38	61.52	61.81	67.43	66.38	63.90	64.80	66.20
Cities	Date									
	1.16	1.17	1.18	1.19	1.20	1.21	1.22	1.23	1.24	1.25
Huangshi	3.84	3.94	4.21	3.75	3.70	3.74	3.86	3.76	3.36	3.42
Shiyan	1.50	1.56	1.65	1.84	1.97	2.00	2.06	2.14	1.96	2.00
Xiangyang	3.44	3.58	3.63	3.81	4.08	4.44	4.50	4.24	3.20	3.41
Yichang	2.48	2.50	2.54	2.69	2.95	3.05	3.18	2.88	2.26	2.79
Jingzhou	6.00	5.93	6.29	6.93	7.29	7.17	7.65	7.31	6.04	5.87
Jingmen	2.81	2.75	2.96	3.31	3.59	3.76	4.19	4.07	3.43	3.32
Ezhou	4.04	4.23	4.39	3.91	3.53	3.28	3.28	3.59	3.99	4.17
Xiaogan	12.57	12.56	13.14	14.47	14.24	13.87	14.56	16.91	17.67	17.01
Huanggang	13.35	14.21	14.87	12.28	12.45	13.50	14.08	14.12	14.58	15.93
Xianning	4.96	5.07	5.14	4.95	4.75	4.77	4.74	4.90	4.48	4.74
Suizhou	2.89	2.98	3.11	3.21	3.38	3.54	3.79	4.15	3.72	3.11
Enshi	1.80	1.74	1.80	1.74	1.87	1.83	1.76	1.54	1.60	2.39
Xiantao	2.80	2.76	2.91	3.07	3.11	3.23	3.30	3.34	3.19	3.38
Tianmen	2.07	1.95	2.10	2.33	2.43	2.28	2.34	2.25	1.94	2.00
Qianjiang	1.03	1.02	1.03	1.04	1.17	1.19	1.35	1.25	0.92	0.90
Shengnongjia	0.10	0.00	0.00	0.10	0.00	0.10	0.00	0.00	0.00	0.00
Total	65.75	66.96	69.96	69.50	70.67	71.48	74.77	76.57	72.46	74.56

Data source: Baidu Migration Big Data Website

## Appendix 2

**Table 4:** Basic information of 16 prefecture-level cities in Hubei Province

	GDP per capita (10,000 CNY)	Distance to Wuhan (km)	Population density (persons/km^2^)	Number of beds (per 1000 persons)	Number of doctors (per 1000 persons)	Date of lockdown
Ezhou	9.33	60.3	676.1	5.64	4.94	1.23
Qianjiang	7.82	137.7	482.0	4.16	4.61	1.23
Xiantao	7.02	88.1	449.2	4.65	4.78	1.23
Enshi	2.58	463.6	140.1	7.14	5.49	1.24
Huanggang	3.22	53.6	362.7	5.67	4.36	1.24
Huangshi	6.42	82.6	539.1	6.37	6.28	1.24
Jingmen	6.38	208.8	234.7	5.81	5.29	1.24
Jingzhou	3.72	201.2	396.4	5.38	4.68	1.24
Xianning	5.36	83.0	253.7	4.74	4.71	1.24
Tianmen	4.65	110.0	485.2	4.72	4.32	1.24
Suizhou	4.56	151.4	230.0	4.45	3.92	1.25
Xiaogan	3.89	49.7	552.2	4.64	3.72	1.25
Xiangyang	7.60	261.6	286.7	6.46	5.23	1.27
Shengnongjia	3.73	367.8	23.6	6.34	5.63	–

Note: *CNY* Chinese Yuan; *km* kilometer. Shengnongjia has not been locked down during this epidemic

## Footnotes

1 Data on January 20th and 21st were excluded because new confirmed cases were reported only in two prefecture-level cities in these two days, which may reduce the statistical power.

2 For the data we used, the diagnosis date is the time one is confirmed to be a COVID-19 patient, not the date when this patient began to have related symptoms. Thus, we should consider the period from contact to being confirmed (contact-infection-latency-onset-diagnosis), and this period will be longer than the incubation period (3–7 days in most cases).

3 The result of Hausman test shows that the random effects model was better than the fixed effect model.
