# Correction to: Influence of population mobility on the novel coronavirus disease (COVID-19) epidemic: based on panel data from Hubei, China

**DOI:** 10.1186/s41256-020-00160-5

**Published:** 2020-06-18

**Authors:** Junfeng Jiang, Lisha Luo

**Affiliations:** 1 School of Health Sciences, Wuhan University, No.115 Donghu Road, Wuhan, 430071 China; 2 Center for Evidence-based and Translational Medicine, Zhongnan Hospital of Wuhan University, Wuhan, China; 3 Department of Evidence-Based Medicine and Clinical Epidemiology, the Second Clinical College of Wuhan University, Wuhan, China

**Correction to: Glob Health Res Policy (2020) 5: 30**

**https://doi.org/10.1186/s41256-020-00151-6**

After publication of this article [1], it is reported that this article contains some errors, details below.

1. The heading “Introduction” should be inserted at the beginning of the article.
2. The section heading “Data and methods” should be changed to “Methods”.
3. The heading “Methods” should be changed to “Data analysis” and it should be a second level heading, the same level with ‘Data sources’ and ‘Variables’.
4. In Results section, the below corrections should be made:
  In the sentence "we used Fig. 3 to display the daily confirmed COVID-19 cases per 10,000 persons in these five prefecture-level cities...", “per 10,000 persons” should be removed.
  In the sentence "The daily increase of diagnosis rate in Huanggang had begun to slowly decline since February 5th", “Huanggang” should be changed to “Xiangyang”.
